# Molecular Surveillance for Potential Zoonotic Pathogens in Troglophilus Bats: Detection and Molecular Characterization of Bat Coronaviruses in Southern Italy

**DOI:** 10.3390/pathogens14050457

**Published:** 2025-05-07

**Authors:** Francesco Mira, Francesca Gucciardi, Giorgia Schiró, Rosario Grasso, Maria Teresa Spena, Gábor Kemenesi, Claudia Vaiana, Davide Anzá, Laura Di Paola, Santina Di Bella, Annalisa Guercio, Giuseppa Purpari

**Affiliations:** 1 Istituto Zooprofilattico Sperimentale della Sicilia “A. Mirri”, 90129 Palermo, Italy; francesca.gucciardi@izssicilia.it (F.G.); claudia.vaiana@gmail.com (C.V.); davide.anza@gmail.com (D.A.); santina.dibella@izssicilia.it (S.D.B.); annalisa.guercio@izssicilia.it (A.G.); giuseppa.purpari@izssicilia.it (G.P.); 2 Department of Veterinary Sciences, University of Messina, Polo Universitario dell’Annunziata, 98168 Messina, Italy; 3 Dipartimento di Scienze Biologiche, Geologiche e Ambientali, Università degli Studi di Catania, 95124 Catania, Italy; rosagra@unict.it (R.G.); spenamariateresa@gmail.com (M.T.S.); 4 National Laboratory of Virology, Szentágothai Research Centre, University of Pécs, H-7624 Pécs, Hungary; 5 Institute of Biology, Faculty of Sciences, University of Pécs, H-7624 Pécs, Hungary

**Keywords:** bat, chiroptera, *alphacoronavirus*, *betacoronavirus*, SARS-CoV-2, COVID-19, zoonoses, Mediterranean basin, Italy

## Abstract

The recent COVID-19 pandemic has renewed interest in bats, as they are natural hosts for numerous viruses, some of which have crossed species boundaries. Despite continued efforts in the past, the ecology of bat-related viruses in a significant part of national territories, such as Italy, remains largely unexplored. Herein, we describe the detection and molecular characterization of bat coronaviruses, identified during a viral survey on selected potential zoonotic pathogens (lyssavirus and coronaviruses) carried out in Sicily, southern Italy. A total number of 330 samples were collected from 149 bats in a period (November 2020–April 2023) overlapping the COVID-19 pandemic. All samples tested negative for lyssavirus and SARS-CoV-2, while 12 bats (8.05%) tested positive to a pan-coronavirus assay. Both alphacoronaviruses and betacoronaviruses were identified in samples from three species (*Miniopterus schreibersii*, *Rhinolophus ferrumequinum*, and *Rhinolophus hipposideros*). Strain sequences were related to coronaviruses detected in the last decade in northern Italy as well as in other countries bordering the Mediterranean basin, suggesting a widespread diffusion of these strains. This study supports the need for further monitoring efforts and early detection of circulating coronavirus genotypes, particularly for those which have been repeatedly emerging as zoonotic spillovers.

## 1. Introduction

Zoonotic diseases, or zoonoses, are diseases shared between animals and people, which commonly spread at the human–animal–environment interface; thus, surveillance systems for their early detection can contribute to supporting coordinated response, prevention, and mitigation measures [1]. Indeed, animals are given a major role as reservoirs of over 75% of emerging infectious human diseases, with wildlife constituting the most significant reservoir of emerging zoonotic diseases [2,3].

The recent COVID-19 pandemic event once again renewed interest in the surveillance of wildlife, particularly of bats, as these animals are considered as a reservoir of coronaviruses (CoVs) and other viruses of the families *Filoviridae*, *Rhabdoviridae* (rabies virus and other lyssaviruses), and *Paramyxoviridae* [4,5,6,7]. Bats (order Chiroptera) comprise 1690 species with a broad geographic distribution and represent the most diverse group of mammals after rodents [8]. The social organization of bats contributes to the maintenance and spread of viruses in the population, particularly in large and mixed species colonies [9,10]. Overall, the crucial role of bats as relevant reservoir hosts and as spillovers for different viruses, as well as the ability for some of these viruses to directly or through intermediate hosts adapt to humans, has been recognized [11,12]. Wildlife or domestic animals act as intermediate hosts for bat-related viruses and their potential role was determined, for example, for the severe acute respiratory syndrome coronavirus 2 (SARS-CoV-2) [13,14,15]. For these reasons, monitoring and early detection of bat-related viruses are of paramount interest in monitoring potential risks from their habitat.

Among the viruses potentially threatening human health, coronaviruses and lyssaviruses have been reported as global concerns. Coronaviruses are enveloped positive-sense RNA viruses, taxonomically included in the order *Nidovirales*, family *Coronaviridae*, and subfamily *Orthocoronavirinae* [16]. Coronaviruses are classified into the four genera *Alphacoronavirus* (α-CoV), *Betacoronavirus* (β-CoV), *Gammacoronavirus*, and *Deltacoronavirus*. All mammalian CoVs, including bat CoVs, belong to the α-CoV and β-CoV genera [17]. All highly pathogenetic human CoVs, such as the severe acute respiratory syndrome coronavirus (SARS-CoV), the Middle East respiratory syndrome coronavirus (MERS-CoV), and the SARS-CoV-2, belong to the β-CoV genus. Previous studies identified in rhinolophid bats (family *Rhinolophidae*, genus *Rhinolophus*) the natural host of SARS-CoV and SARS-like CoVs [17,18,19,20,21]. Some studies have also supposed the potential role of *Rhinolophus ferrumequinum*, present in Italy, as a potential reservoir for SARS-CoV-2 [22,23,24].

The genus *Lyssavirus* is taxonomically included in the *Alpharhabdovirinae* subfamily, *Rhabdoviridae* family, and includes negative-sense RNA viruses, subdivided into two phylogroups (phylogroup I includes *Rabies Virus*, RABV, responsible for the vast majority of rabies cases worldwide) and some divergent viruses in the genus (such as the *West Caucasian Bat Lyssavirus*, WCBV), which are not members of either of these phylogroups [25,26]. Lyssaviruses are essentially neurotropic pathogens, which found in bats the principal worldwide reservoir hosts, whereas the circulation of RABV is maintained by carnivores (order Carnivora) as well as bats [26,27,28]. WCBV has been described in the *Miniopterus schreibersii*, suggesting a potential role of the genus *Miniopterus* in the evolution of divergent lyssaviruses, and spillover events have been reported in domestic cats [25]. The recent evidence of further spillover events in cats, involving the European bat lyssavirus types 1 [29], closely related to classical RABV and responsible for an emerging zoonosis [30], further strengthen the need for monitoring. According to the current Regulation (EU) 2016/429 of the European Parliament and of the Council of 9 March 2016 (“Animal Health Law”) and the related Commission Implementing Regulation (EU) 2018/1882 of 3 December 2018, surveillance for RABV in the Order chiroptera (“category E disease”) is currently implemented in Italy.

Between 1980 and 2002, 34 bat species have been in Italy, belonging to the *Rhinolophidae*, *Vespertilionidae*, *Miniopteridae*, and *Molossidae* families [31], while a total of 28 bat species have been reported in Sicily, the largest island in the Mediterranean Sea [32,33,34,35,36]. Sicily is the southernmost region of Italy, located in the center of the western Mediterranean Sea, spanning over 25,711 Km^2^, with a typical Mediterranean climate and a diverse landscape, spanning from coastlines to mountains and volcanoes, with protected areas, sedentary or migrating fauna, and large human settlements, thus representing one of the most significant biodiversity hotspots in the Mediterranean basin. Moreover, most of these bat species are also common in North African countries bordering the western Mediterranean [37], geographically shortly distant to Sicily.

Several previous studies reported the geographical and temporal distribution of CoVs in different bat species, either in urban/sub-urban or in rural areas and caves, but a complete global assessment of bat coronavirus infection still deserves to be defined. Particularly, a clearer definition of bat coronaviruses in the Mediterranean basin area is thought necessary. Most European studies, specifically referring to those on CoVs in bats, were conducted in Italy, almost all focusing in northern or central Italy with some exceptions for those conducted in Sardinia, a major island, and a few in southern Italy [38,39,40,41,42].

The objective of this study, covering the years 2020–2023 and overlapping almost all of the COVID-19 pandemic, was to conduct a prospective sampling and testing to investigate the presence and genetic features of selected viruses, specifically lyssaviruses and coronaviruses, in bats from caves in Sicily, southern Italy. The aim of this study was to evaluate the occurrence and diversity of these viruses in a geographical area to date lacking specific data, contributing to better understanding their circulation patterns and the related bat species hosts.

## 2. Materials and Methods

### 2.1. Sampling Location and Samples Collection

**Figure 1:**
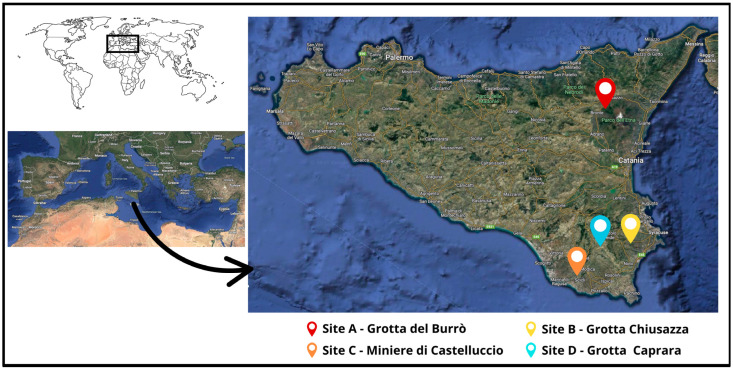
Geographical location of the caves described in this study. Physical maps were obtained from Google Earth (Google Landsat/Copernicus Data SIO, NOAA, U.S. Navy, NGA, GEBCOInst. Geogr. NacionalGeoBasis-DE/BKG (©2009) Mapa GISrael).

Bat sampling was carried out between 15 November 2020 and 27 April 2023, at four bat caves (namely grotta del Burrò, grotta Chiusazza, miniere di Castelluccio, and grotta Caprara) with different locations in eastern Sicily, southern Italy (Table 1 and Figure 1). These caves are separated by a minimum of 25.97 (caves B and D) and maximum of 109.78 (caves A and C) kilometers. Details on the caves and their bat populations were reported in a related study [43].

During five separate sampling dates (site A on 15 November 2020; site B on 16 March 2022 and 5 December 2022; site C on 29 March 2023; site D on 27 April 2023), bats were captured for the sample collection by using hand nets, handled, identified (based on morphological identification), and sampled by expert and authorized personnel (R.G. and M.T.S.), according to current legislation and complying with the guidelines for the monitoring of chiroptera drawn up by the Istituto Nazionale Fauna Selvatica, Italy [31].

A total number of 148 bats, belonging to 3 families (*Miniopteridae*, *Vespertilionidae*, and *Rhinolophoidae*) and 6 species (*Miniopterus schreibersii*, *Myotis capaccinii*, *Myotis myotis*, *Rhinolophus euryale*, *Rhinolophus ferrumequinum*, and *Rhinolophus hipposideros*), were captured. All bats were released at the site of capture immediately after sample collection. In addition, tissue samples (brain, heart, lungs, liver, kidney, and intestine) were obtained from a dead bat (*Miniopterus schreibersii*) in good post-mortem conditions found during the sampling at site A.

A total number of 330 samples (n = 148 oral swabs, n = 66 urines, n = 86 feces and n = 24 rectal swabs, n = 6 tissue samples) were collected from the captured bats and the single dead bat (more details in Supplementary Table S1). As collected, some urine and feces were conferred to the laboratory as a unique mixed sample. Collected samples were rapidly stored at −20 °C, transported to the laboratory, and stored at −80 °C until further processing.

### 2.2. Samples Processing

A total volume of 2 mL of a culture medium (Eagle’s Minimum Essential Medium (EMEM); Sigma–Aldrich^®^, Milan, Italy) supplemented with an antibiotic and antimycotic solution 10× (1000 U/mL penicillin G sodium salt, 1 mg/mL streptomycin sulfate, 2.5 μg/mL amphotericin B; Euro Clone^®^, Milan, Italy) was added to each swab. Swabs were then maintained at +37 °C for 60 min, and finally briefly vortexed. Swab suspensions were transferred into a 15 mL sterile tube, centrifuged at 1500× *g* for 15 min at +4 °C, and then supernatants were finally transferred to sterile labeled bijoux and stored at −80 °C.

Fecal, urine, and organ tissue samples were homogenized (10% *w*/*v*) in a culture medium (Eagle’s Minimum Essential Medium (EMEM); Sigma–Aldrich^®^, Milan, Italy) supplemented with an antibiotic and antimycotic solution 10× (1000 U/mL penicillin G sodium salt, 1 mg/mL streptomycin sulfate, 2.5 μg/mL amphotericin B; Euro Clone^®^, Milan, Italy). Homogenates were then centrifuged at 1500× *g* for 15 min at +4 °C and supernatants were transferred to sterile labeled bijoux and stored at −80 °C.

The RNA extraction was conducted from 140 μL of each supernatant by using the QIAamp Viral RNA Mini Kit (Qiagen S.p.A., Hilden, Germany), according to the manufacturer’s instructions, and nucleic acids were stored at −80 °C until processed.

### 2.3. Virus Screening

Screening for lyssaviruses, coronaviruses, and SARS-CoV-2 was conducted using different nested reverse transcription (RT)-PCR or real-time RT-PCR protocols. The results were reported as detection frequencies and their relative confidence intervals, calculated using the binomial Clopper-Pearson exact method.

#### 2.3.1. Lyssavirus Screening

A preliminary one-step real-time RT-PCR assay, using primers and probes targeting the highly conserved non-coding leader region and part of the nucleoprotein (N) coding sequence of the Lyssavirus genome [44,45], was performed. Real-time PCR assays were carried out in a QuantStudio^™^ 6 Flex Real-Time PCR System (Applied Biosystems^™^, Thermo Fisher Scientific, Carlsbad, CA, USA). Briefly, the detection was carried out using the AgPath-ID^™^ One-Step RT-PCR Reagents (Applied Biosystems^™^, Thermo Fisher Scientific, Carlsbad, CA, USA) in a 25 μL reaction mix consisting of the following: 12.5 μL of 2X RT-PCR Buffer, 1 μL of (20 μM) pre-mix of primers LN34 Forward Primer 1 and LN34 Forward Primer 2, 1 μL of (20 μM) primer LN34 Reverse Primer, 1 μL of (5 μM) pre-mix probes LN34 (LNN34 and LN34lago) (more details of primers and probe were provided in Table 2), 1 μL of 25X RT-PCR Enzyme Mix, 2 μL of RNA extract, and 6.5 μL of nuclease-free water.

A parallel one-step real-time RT-PCR assay was performed to amplify and detect host β-actin mRNA [44]. Briefly, the amplification was carried out using the AgPath-ID™ One-Step RT-PCR Reagents (Applied Biosystems^™^, Thermo Fisher Scientific, Carlsbad, CA, USA) in a 25 μL reaction mix consisting of the following: 12.5 μL of 2X RT-PCR Buffer, 1 μL of (10 μM) primer β-Actin Forward Primer, 1 μL of (10 μM) β-Actin Reverse Primer, 1 μL of (5 μM) β-Actin Probe (details in Table 2), 1 μL of 25X RT-PCR Enzyme Mix, 2 μL of RNA extract, and 6.5 μL of nuclease-free water.

Both amplifications were conducted under the following thermal conditions: 50 °C for 30 min and 95 °C for 10 min, followed by 45 cycles at 95 °C for 15 s, and 56 °C for 30 s. A viral strain (brain material experimentally infected with rabies virus strain CVS11) obtained from the National Reference and United Nations FAO Reference center and WOAH Reference Laboratory for rabies at the Istituto Zooprofilattico Sperimentale delle Venezie (IZSVe), Padua, Italy, and nuclease-free water were used as positive and negative controls, respectively.

#### 2.3.2. Coronavirus Screening

A set of broad range RT-nPCR and Real-Time RT-PCR assays were performed to detect coronaviruses and specifically target SARS-CoV-2, respectively.

The presence of coronavirus (CoV) RNA was evaluated using a broadly reactive (pan-CoV) set of primers [46], developed by a combination of existing primers from different studies [47,48,49], targeting the RNA-dependent RNA polymerase (RdRp) (Table 2). Nested PCR assays were carried out in a SimpliAmp^™^ thermal cycler (Applied Biosystem, Thermo Fisher Scientific Inc., Waltham, MA, USA). Reverse transcription and first round amplification (pan-CoV first) were carried out in a one-step protocol using the QIAGEN^®^ OneStep RT-PCR Kit (Qiagen S.p.A, Hilden, Germany) in a 25 μL mix containing 5 µL of 5x QIAGEN OneStep RT-PCR Buffer, 2.5 µL of each primer (10 μM) Hu-F and Hu-R, 1 µL of (10 mM) dNTP Mix, 0.25 μL of RNase Inhibitor (40 U/μL; Euroclone S.p.A., Pero, Italy), 5 μL of RNA extract, and 7.75 µL of nuclease-free water. The reaction was conducted under the following thermal conditions: 50 °C for 30 min and 95 °C for 15 min, followed by 35 cycles of 94 °C for 20 s, 52 °C for 40 s, 72 °C for 60 s, and a final extension of 72 °C for 10 min.

For the second round (pan-CoV nested), 1 μL of the first amplicon was used as input in a PCR reaction using the Platinum^™^ Taq DNA Polymerase (Invitrogen, Thermo Fisher Scientific, Carlsbad, CA, USA) in a 25 μL mix containing 2.5 µL of 10X PCR Buffer, 1 µL of each primer (10 μM) Poon-F and Chu06-R1, 0.5 µL of (10 mM) dNTP Mix, 2.5 μL of MgCl_2_ (25 mM/μL; Applied Biosystem, Thermo Fisher Scientific, Carlsbad, CA, USA), 0.2 μL of PlatinumTM Taq DNA Polymerase, and 16.3 µL of nuclease-free water. The reaction was conducted under the following thermal conditions: 94 °C for 3 min, followed by 30 cycles of 94 °C for 20 s, 50 °C for 30 s, and 72 °C for 50 s. A canine coronavirus RNA (canine coronavirus, ATCC 809-VR) and nuclease-free water were used as positive and negative controls, respectively, in all reactions.

The presence of SARS-CoV-2 RNA was evaluated with a real-time RT-PCR assay by using the commercial kit TaqPath^™^ COVID-19 CE-IVD RT-PCR Kit (Applied Biosystems^™^). Briefly, 5 μL of RNA extract was used in a 25 μL mix containing 6.25 µL of TaqPath^™^ 1-Step Multiplex Master Mix (No ROX^™^) (4X), 1.25 µL of COVID-19 Real-Time PCR Assay Multiplex, and 12.5 µL of nuclease-free water. The reaction was conducted under the following thermal conditions: 25 °C for 2 min, 53 °C for 10 min, and 95 °C for 2 min, followed by 40 cycles of 95 °C for 3 s and 60 °C for 30 s. Positive and negative controls, as well as the MS2 Phage control, all provided with the kit, were added in all reactions.

### 2.4. Sequence and Phylogenetic Analyses

PCR amplicons were purified with Illustra^™^ GFX^™^ PCR DNA and Gel Band Purification Kit (GE Healthcare Life Sciences, Amersham, Buckinghamshire, UK) and submitted to BMR Genomics srl (Padua, Italy) for direct Sanger sequencing in both directions.

Overlapping sequences were assembled to obtain a partial RdRp sequence (440 base pairs long), and analyzed using Geneious Prime 2022.0.2 (Biomatters, San Diego, CA, USA). Sequence data were submitted to the GenBank databases under accession numbers PV392527-PV392538. Nucleotide sequence similarities and statistical significance of matches were inferred using the Basic Local Alignment Search Tool (BLAST) [50] (https://blast.ncbi.nlm.nih.gov/Blast.cgi, accessed on 25 May 2024), using the default values to find homologous hits.

A partial CoV RdRp gene sequence dataset (275 reference strain sequences, including alphacoronavirus—HCoV-229E, HCoV-NL63—and betacoronavirus—HcoV-HKU1, HCoV-OC43, MERS-CoV, SARS-CoV, SARS-CoV-2—human CoV sequences [51]) was generated by searching the nucleotide BLAST (BLASTn) tool (accessed on 24 June 2024) and related sequences were downloaded in FASTA format. The associated metadata (accession number, country and year of strain collection, host species, and strain name) for each sequence were registered. Phylogenetic analysis was carried out by comparing the homologous sequences of the related viral strains, aligning them using a dedicated software (Geneious Prime 2022.0.2). The alignment was then fed to MEGA X software [52] to build a Maximum Likelihood (ML) phylogenetic tree selecting the substitution model with the lowest Bayesian Information Criterion (BIC) (General Time Reversible with Gamma distribution and Invariant sites; GTR + G + I) and bootstrap analyses with 1000 replicates.

## 3. Results

### 3.1. Virus Detection

From November 2020 to April 2023, a total of n = 148 oral swabs, n = 66 urines, n = 86 feces and n = 24 rectal swabs, and n = 6 tissue samples collected from 149 bats were analyzed to detect lyssaviruses, coronaviruses, and SARS-CoV-2. Both assays targeting the lyssavirus and SARS-CoV-2 RNA tested negative, excluding the presence of these viruses.

Out of the n = 152 urine/feces and n = 24 rectal swabs samples collected in all four sites, 8 (5.26%; 95CI: 2.3–10.11%) and 5 (20.83%; 95CI: 7.13–42.15%) samples tested positive to the pan-CoV assay, respectively. All other samples tested negative. Interestingly, only the urine, feces, and rectal swab samples yielded positive results, compared to the other samples (oral swabs and tissue samples). Coronavirus-positive samples were detected at sites A (n = 1), B (n = 4), and C (n = 8), while all samples collected at site B on 16th March 2022 and at site D gave negative results.

The 13 positive samples were collected from 12 bats (two different samples collected from the same animal id. IZSSI_2023PA9623idRin23 tested positive) (8.05% of tested bats; 95CI: 4.23–13.65%). Different detection frequencies were observed in sampled bat from each cave: from caves A, B, and C, a 5% (95CI: 0.13–24.87%), 5.06% (95CI: 1.4–12.46%), and 29.16% (95CI: 12.62–51.09%) detection rate, respectively, was determined. Positive results were obtained from samples collected from three species, detailed in Table 3, with different overall detection rates: 5.49% (95CI: 1.81–12.36%) for *Miniopterus schreibersii*, 14.28% (95CI: 5.43–28.54%) for *Rhinolophus ferrumequinum*, and 7.14% (95CI: 0.18–33.87%) for *Rhinolophus hipposideros*. Details on coronavirus-positive samples are provided in Table 3.

### 3.2. Coronavirus Sequence and Phylogenetic Analyses

All but one amplicon obtained from the CCoV screening was successfully sequenced; a positive sample (id. IZSSI_2023PA9623idRin21) was then excluded from subsequent analyses because of the poor-quality raw reads. A total of 12 partial RdRp sequences (two obtained from the same bat) were thus typed according to the BLAST results, revealing the presence of n = 5 alphacoronaviruses (in 2.68% of tested bats; 95CI: 1.1–7.66%) and n = 7 betacoronaviruses (in 4.69% of tested bats; 95CI: 1.91–9.44%) (Table 4). While alphacoronaviruses were only detected in sites A and B, all the betacoronaviruses were detected only in site C.

The analyzed RdRp partial sequences showed a pairwise nucleotide (nt) identity ranging between 62.7% and 100%: in detail, sequences were grouped according to the nt identity into three groups, identified as group 1 (including Alphacoronavirus strains id. IZSSI_2021PA49981idMin6 and IZSSI_2023PA3616idMin39), group 2 (Alphacoronavirus strains id. IZSSI_2023PA3616idMin13, IZSSI_2023PA3616idMin20, and IZSSI_2023PA3616idMin27), and group 3 (Betacoronavirus strains id. IZSSI_2023PA9623idRin23r, IZSSI_2023PA9623idRin23u, IZSSI_2023PA9623idRin14, IZSSI_2023PA9623idRin2, IZSSI_2023PA9623idRin7, IZSSI_2023PA9623idRin10, and IZSSI_2023PA9623idRin11). Each group showed high intra-group pairwise nt identity (group 1: 99%; groups 2 and 3: 100%) and different inter-groups nt identities (group 1 vs. 2: 75–74.7%; group 1 vs. 3: 67.5–67%; group 2 vs. 3: 62.7%). Alphacoronavirus strains id. IZSSI_2021PA49981idMin6 and IZSSI_2023PA3616idMin39 were detected from the two different sites A (year 2020) and B (year 2022), respectively. Moreover, all alphacoronaviruses were detected only in *Miniopterus schreibersii*, while betacoronaviruses were only detected in *Rhinolophus ferrumequinum* and *Rhinolophus hipposideros*.

Group 1 showed the highest nucleotide identities with the sequences of Alphacoronaviruses detected in Portugal in 2022 (99.73–99.46%) from a *Myotis myotis* (accession number OQ613368) and a *Miniopterus schreibersii* (acc.nr. OQ613367), and in central Italy in 2021 (99.55–99.09%) from two *Miniopterus schreibersii* (acc.nrs. ON834690 and OP627105). Slightly lower nt identities, ranging between 99.49% and 98.43%, were observed with Alphacoronavirus detected in France in 2014 (acc.nr. KY423482), Lebanon in 2020 (acc.nr. MW880977), and Bulgaria in 2008 (acc.nrs. GU190242, GU190246, and GU190247), all from two *Miniopterus schreibersii*.

Group 2 showed the highest nucleotide identities with the sequences of Alphacoronaviruses detected in northwestern Italy in 2016 (97.05–96.82%) from three *Rhinolophus ferrumequinum* (acc.nrs. KY780388, KY780389, KY780390). A slightly lower nt identity was observed with alphacoronaviruses detected (96.73%) from a *Rhinolophus ferrumequinum* (acc.nr. KJ652329) and (96.13%) from a *Rhinolophus hipposideros* (acc.nr. KJ652330), both sampled in Hungary in 2013.

Group 3 showed the highest nucleotide identities with the sequences of Betacoronaviruses detected in northwestern Italy in 2016 (98.36–96.34%) from three *Rhinolophus ferrumequinum* (acc.nrs. KY780394, KY780399, and KY780400). Slightly lower nt identities were observed with betacoronavirus detected in Northwest Italy in 2009 (97.66–97.36%; acc.nrs. KC633198, KC633199, and KC633200), in the Sardinia region (Italy) in 2015/2016 (96.57%; acc.nr. MG975784), and in Northwest Italy in 2016 (acc.nr. KY780391), all from *Rhinolophus ferrumequinum*, as well as from a *Rhinolophus ferrumequinum* and a *Rhinolophus euryale* in Bulgaria in 2008 (96.07% and 95.81%, respectively; acc.nrs. GU190231 and GU190224).

Moreover, this group of sequences of betacoronaviruses detected in Sicily showed a lower nucleotide identity (88.38%) with the SARS-CoV strain Tor2 (NCBI Reference Sequence: NC_004718.3), isolated from a human patient in Toronto, Canada, and (87.30%) with the SARS-CoV-2 strain Wuhan-Hu-1 (NCBI Reference Sequence: NC_045512.2), isolated from a human in December 2019 in China. Further lower nucleotide identity rates were shown with the HuCoVs strains HcoV-HKU1, HCoV-OC43, and MERS-CoV (68.4–64%), and with the MERS-CoV-related betacoronavirus strains detected in Northern Italy (acc.nrs. KF500950, MG596803, KF500948, KF500947, KF500946, KF500951, KF500943, KF500944, KF500940, KF500942, MG59680, KY780396, KF500941, KF312399) (66.1–64.6%).

**Figure 2:**
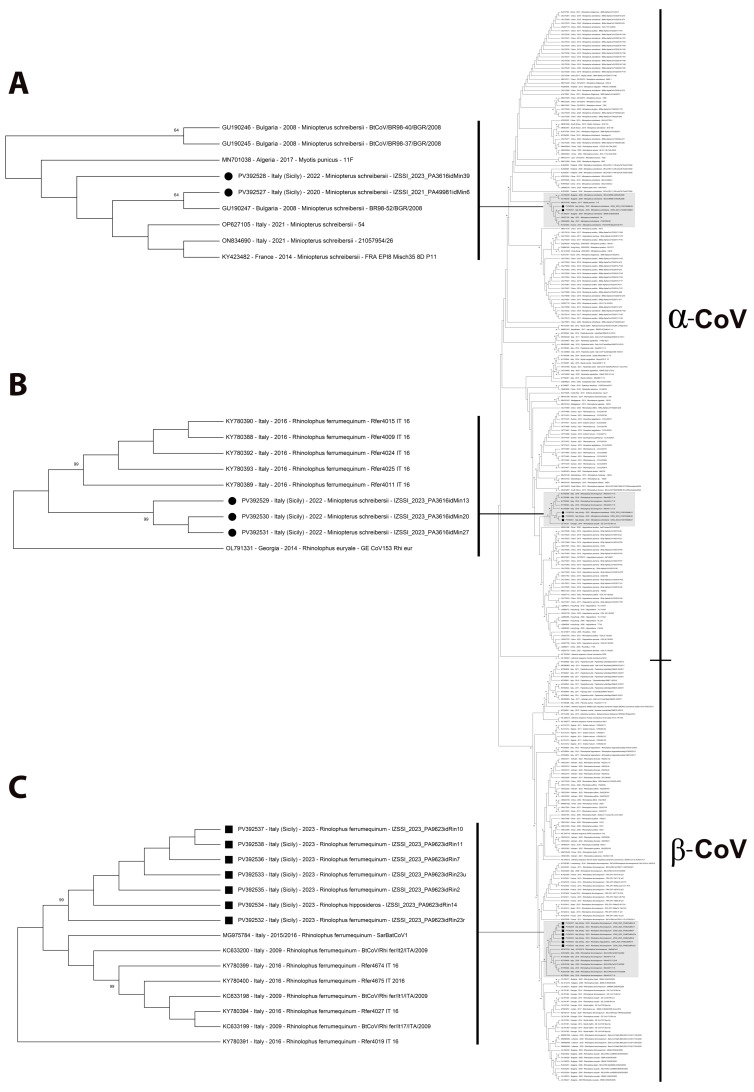
Maximum Likelihood (ML) tree (on the right) based on 287 partial RNA-dependent RNA polymerase (RdRp) gene sequences of coronaviruses (bootstrap values greater than 60 are shown). The ML tree is also depicted as Supplementary Materials Figure S1. The branches, including sequences of coronavirus strain detected in this study, are highlighted in gray and drawn to scale (on the left, as sub-clades (**A**), (**B**), and (**C**), respectively): black dot markings (●) indicate alphacoronavirus strains, while black squared markings (■) indicate betacoronavirus ones, respectively.

As shown in Figure 2, partial RdRp sequences clustered into two main groups, including alphacoronaviruses and betacoronaviruses, respectively (also in Supplementary Materials Figure S1), and viral sequences obtained with this study are included in three separate sub-clades within the alphacoronavirus (groups 1 and 2 in sub-clades A and B, respectively) and betacoronavirus (group 3 in sub-clade C) groups (highlighted in gray and drawn to scale in Figure 2).

## 4. Discussion

The recent COVID-19 pandemic renewed once again the interest in the diversity and distribution of circulating bat-related viruses. As bats have been considered reservoirs of several viruses potentially threatening human and animal health [53,54,55], understanding the natural diversity and ecological patterns of these viruses has become an important research area once again. As a recognized natural host of a rich and diverse gene pool of viruses, this surveillance can contribute to depicting and updating bat-borne virus ecology, contributing to supporting global outbreak prevention efforts.

Considering human coronaviruses (HCoVs), it is generally accepted that SARS-CoV, MERS-CoV, HCoV-229E, HCoV-NL63, and SARS-CoV-2 have a direct or intermediate host-derived origin from the bat coronavirus genetic pool [56,57]. Other human coronaviruses (HCoV-OC43 and HCoV-HKU1) have closer evolutionary links to rodent-borne CoVs [58]. Moreover, novel members of the sarbecovirus genus with the ability of crossing species barriers were discovered in Russia [59], and more recently a novel virus from the merbecovirus group was reported in China [60]. Thus, active surveillance could lead to mapping the local viral population of this species, necessary for subsequent evaluations based on their current circulation in bat populations.

Although intense investigation on bat-related viruses has significantly increased in the last few years following the COVID-19 pandemic, the global mapping of selected viruses and their distribution in some geographic areas still remains to be determined in-depth. Indeed, the relationship between viruses and their hosts in comparison to the geographical area of detection continues to be debated [61]; thus, the evidence-based mappings continue to receive specific attention.

To contribute to this direction, this study analyzed samples collected from bats in Sicily, the southernmost region of Italy, located in the center of the western Mediterranean Sea, a geographic interface between Europe and Africa. In the recent past, only one study that reported the sample collection from this region tested for bat coronaviruses, even if with negative results [62]. Therefore, this study represents the first and updated structured study to detect bat viruses in a potential critical geographic area such as Sicily. Moreover, this study overlaps the COVID-19 pandemic period, aiming to add specific data on coronaviruses in bats.

Based on the obtained data, only coronavirus-positive results were obtained. Indeed, all samples tested negative both for lyssaviruses and for the SARS-CoV-2. This result is not surprising, since a certain degree of endemism of coronaviruses in this species is widely recognized, considering the continuous detection in almost all previous studies in Italy [17,39,40,41,62,63,64,65], although SARS-CoV-2 has not yet been detected in bats during previous Italian monitoring studies [39,42]. Conversely, less positive results for lyssaviruses in bats, as in a domestic cat, were reported in Italy, also considering the lack of specific studies, except for some relevant ones [25,54,66,67].

An 8.05% of CoV positivity rate was registered, and this result is similar to the rates obtained in other studies on Italian bat populations, ranging between 2.7% [62] and 12% [64] (Table 5; average value referred to Italian studies: 6.8%). Unfortunately, the lack of other more recent studies on bat CoVs in Italy or previous studies from the same regional area prevented further comparison on the epidemiology of these viruses. Despite all these previous studies that screened bat CoV RNA targeting the RdRp genomic region and considered the intrinsic genomic diversity of CoVs, differences in the detection rates in Italy could be also related to the specificity of the used primer sets, as also observed by [68]; thus, further comparisons are necessary to exclude this hypothesis. For this reason, a validated broad-range pan-coronavirus assay [46] was preferred for the study design. In addition to methodological differences, the seasonal variation of CoV prevalence may also affect the comparability between studies [69].

This study was conducted on samples collected from six bat species which are distributed in but not limited to wide European areas, showing ecological features facilitating the chances of virus spread, some of which are sedentary or short-distance seasonal migrant [12,20,70,71,72]. As some species (such as *Miniopterus schreibersii*) are mostly cave-dwelling, they are rarely found by human activities to be included for specific surveillances for bat-harbored viruses, such as lyssaviruses [55]. Among these species, *Rhinolophus ferrumequinum* has been reported to be associated with SARS-like CoVs [17,18,19,20,21].

To date, alphacoronaviruses and betacoronaviruses have been reported in Italy in several bat species. Generally, *Vespertilionidae*, *Rhinolophidae*, and *Miniopteridae* harbor both α-CoVs and β-CoVs in Europe, and the link between CoV genera and host species contribute to detect interhost-switching events, an important driver of viral evolution [73]. With this study, positive results were found in samples from *Miniopterus schreibersii* for alphacoronaviruses, and from *Rhinolophus ferrumequinum* and only one *Rhinolophus hipposideros* for betacoronaviruses.

Alphacoronaviruses in *Miniopterus schreibersii* have been previously reported in other European countries such as Bulgaria in 2008 [20], France and Spain in 2008–2016 [21], Portugal in 2022 [38], or Lebanon in 2020 [12], but not in Italy yet, with the exception of two bat alphacoronavirus sequences obtained from the GenBank database (accession numbers ON834690 and OP627105), originated from central Italy in 2021 [unpublished data]. Except for these latter genomic data, the data obtained from this study represent the first description of alphacoronaviruses detected in *Miniopterus schreibersii* bats in Italy. These strains originated from two different caves (caves A and B, 89.52 km distant), detected in different timeframes (years 2020 and 2022). Differently, other bat α-CoVs, herein described in group 2 and detected in the same above cited cave B, were related to the α-CoVs detected mainly in *Rhinolophus ferrumequinum* and *Rhinolophus hipposideros* (fam. *Rhinolophidae*) in Italy in 2016 [64] and in Hungary in 2013 **[68]**. These data do not substantially contribute to solve the still open debate on the relationships between the bat coronavirus genera and the host species or the geographic origin; differently to the early studies which observed a likely relationship with the host species to predict geographic distribution [20,64], the most recent ones did not find this univocal relationship, which is more likely related to the co-evolution of the virus in the animal host and the social structure of the bat colonies [38]. As in this study, the detection of bat CoVs of the same genera from different host species, from the same or different caves, is prone to lean towards these latter hypotheses.

The betacoronaviruses detected in this study only from *Rhinolophus ferrumequinum* and *Rhinolophus hipposideros* were related to those detected in *Rhinolophidae* from Sardinia in 2015–2016 [40], from Northwestern Italy in 2009 and 2016 [17,64], and from Bulgaria in 2008 [20]. The only one previous description of bat betacoronaviruses in *Rhinolophus hipposideros* from Italy reports their detection in 2011–2012 again in northern regions [63]. This further CoV strain detected in *Rhinolophus hipposideros* from Italy showed a closer relationship with those from northern Italy or east Europe, but was detected in *Rhinolophus ferrumequinum*. A comprehensive resuming of the previous Italian studies on bat coronaviruses and of the bat coronavirus strains related to this study is proposed in Table 5.

The positive results yielded only in urines, feces, and rectal swabs align with other studies in which higher detection rates were found in rectal swabs and stools [12,38], being this occurrence related to the most likely viral excretion route or to test sensitivity. Moreover, it cannot be excluded that the single time of collection, as in this study, could also have conditioned this observation. Nonetheless, these data suggest feces as an appropriate biological matrix to monitor CoV spread in bats, reducing their manipulation, as also observed in [64].

Some limitations of this study should be acknowledged as a wider description was limited by (i) the temporal discontinuity in the detection, (ii) the detection limited to caves and their specific bat populations, (iii) the limited panel of virus detection, or (iv) the lack of whole genome sequencing or, at least, of the Spike gene sequencing.

First, as viral excretion cannot be continuous or could be influenced by the viral titer, the obtained results could have been influenced by the different timeframes of collection in the different caves. Moreover, this study was limited to bats in caves not including other urban/suburban contexts or anthropogenic activities. Bats’ habits and roosts can be different, and these various conditions can contribute to determining the risks for bat–human transmissions of pathogens or risk perception [74].

The limitations in the study design should also be acknowledged; despite the potential bat-related viruses threatening human health, only lyssavirus and coronavirus were tested. It is undeniable that the opportunity to collect samples from this species offers the opportunity to extend the studies to other viral targets. Moreover, the CoV detection and typing were based on the RdRp genomic region, even if an additional contribution could have been obtained by the spike gene sequencing or, preferably, by the whole gene sequencing. Nonetheless, the study design warranted comparable data for our description.

In conclusion, this study represents the first report of CoV genetic data in bats from southern Italy and probably the most southern European study, as a bridge with Mediterranean basin countries. Altogether, these results depict the Italian territories as apparently highly geographical different, showing a certain degree of virus diversity within the same country, and demonstrate how the alphacoronaviruses and betacoronaviruses already circulating in European and Mediterranean bats before the COVID-19 pandemic event still persist at least within the tested regional bat population. Moreover, despite the epidemic in humans, SARS-CoV-2 likely did not impact the bats in the tested areas, and the detected strains show a species-specific segregation. To confirm these data, further surveillance studies are necessary within Italy and, more widely, in the Mediterranean basin. This would potentially lead to better understanding of the complexity of coronavirus ecology and evolution at the bordering region of Africa and Europe.

## Figures and Tables

**Table 1:** Sampling sites.

Site	Site Name	Geographical Location City (Province)	Geographic Coordinates (Longitude—Latitude)	Number of Sampled Bats
A	Grotta del Burrò	Randazzo (CT)	14.934538–37.826880	21
B	Grotta Chiusazza	Floridia (SR)	15.159536–37.026406	38 (on 16 March 2022) 41 (on 5 December 2022)
C	Miniere di Castelluccio	Modica (RG)	14.691320–36.837110	24
D	Grotta Caprara	Noto (SR)	14.926690–37.007520	26
			Total sampled bats:	150

**Table 2:** Primers and probes used for the virus screening real-time RT-PCR and RT-PCR assays.

PCR assay	Target	Primers/Probes	Sequence (5′-3′)	Reference
Real-time Lyssavirus	highly conserved non-coding leader region and part of the nucleoprotein (N) coding sequence	LN34 Forward Primer 1	ACGCTTAACAACCAGATCAAAGAA	[44]
		LN34 Forward Primer 2	ACGCTTAACAACAAAATCADAGAAG	
		LN34 Reverse Primers	CMGGGTAYTTRTAYTCATAYTGRTC	
		LN34 Probe	(FAM)-AA+C+ACCY+C+T+ACA+A+TGGA-(BHQ1)	
		LN34lago Probe ^a^	(FAM)-AA+C+ACTA+C+T+ACA+A+TGGA-(BHQ1)	
Real-time host β-actin mRNA	β-actin mRNA	β-actin Forward Primer	CGATGAAGATCAAGATCATTGC	
		β-actin Reverse Primer	AAGCATTTGCGGTGGAC	
		β-actin Probe	(HEX)-TCCACCTTCCAGCAGATGTGGATCA-(BHQ1)	
Nested PCR pan-coronavirus	RNA dependent RNA polymerase (RdRp)	Hu-F	AARTTYTAYGGHHHYTGG	[46]
		Hu-R	GARCARAATTCATGHGGDCC	
		Poon-F	GGTTGGGACTATCCTAAGTGTGA	
		Chu06-R1	CCATCATCAGATAGAATCATCAT	

^a^ The reference for the LN34lago probe is [45].

**Table 3:** Details on coronavirus-positive samples.

Collection Date (Day Month Year)	Site	Detection Frequency	Host Species	Sample	Id
15 November 2020	A	4.76%	*Miniopterus schreibersii*	Feces	IZSSI_2021PA49981idMin6
5 December 2020	B	5.06%	*Miniopterus schreibersii*	Urine/Feces	IZSSI_2023PA3616idMin39
			*Miniopterus schreibersii*	Urine/Feces	IZSSI_2023PA3616idMin13
			*Miniopterus schreibersii*	Urine/Feces	IZSSI_2023PA3616idMin20
			*Miniopterus schreibersii*	Urine/Feces	IZSSI_2023PA3616idMin27
29 March 2023	C	29.16%	*Rhinolophus ferrumequinum* ^a^	Rectal swab	IZSSI_2023PA9623idRin23r
			*Rhinolophus ferrumequinum* ^a^	Urine	IZSSI_2023PA9623idRin23u
			*Rhinolophus hipposideros*	Urine	IZSSI_2023PA9623idRin14
			*Rhinolophus ferrumequinum*	Rectal swab	IZSSI_2023PA9623idRin2
			*Rhinolophus ferrumequinum*	Rectal swab	IZSSI_2023PA9623idRin7
			*Rhinolophus ferrumequinum*	Rectal swab	IZSSI_2023PA9623idRin10
			*Rhinolophus ferrumequinum*	Rectal swab	IZSSI_2023PA9623idRin11
			*Rhinolophus ferrumequinum*	Urine	IZSSI_2023PA9623idRin21

^a^ Collected from the same animal.

**Table 4:** Details on coronavirus sequences obtained in this study.

Sequence Id	Accession Number	Genera	Site	Host Species
IZSSI_2021PA49981idMin6	PV392527	Alphacoronavirus	A	*Miniopterus schreibersii*
IZSSI_2023PA3616idMin39	PV392528		B	*Miniopterus schreibersii*
IZSSI_2023PA3616idMin13	PV392529			*Miniopterus schreibersii*
IZSSI_2023PA3616idMin20	PV392530			*Miniopterus schreibersii*
IZSSI_2023PA3616idMin27	PV392531			*Miniopterus schreibersii*
IZSSI_2023PA9623idRin23r	PV392532	Betacoronavirus	C	*Rhinolophus ferrumequinum* ^a^
IZSSI_2023PA9623idRin23u	PV392533			*Rhinolophus ferrumequinum* ^a^
IZSSI_2023PA9623idRin14	PV392534			*Rhinolophus hipposideros*
IZSSI_2023PA9623idRin2	PV392535			*Rhinolophus ferrumequinum*
IZSSI_2023PA9623idRin7	PV392536			*Rhinolophus ferrumequinum*
IZSSI_2023PA9623idRin10	PV392537			*Rhinolophus ferrumequinum*
IZSSI_2023PA9623idRin11	PV392538			*Rhinolophus ferrumequinum*

^a^ Collected from the same animal.

**Table 5:** Resume of studies that sampled CoVs in bats in Italy, and in countries across Europe and bordering the Mediterranean Sea (the latter related to this study). Country (and area for the Italian studies) and year of collection, identified CoV genera, positive bat species, and positivity rates were included in the table.

Country (Area) and Year of Collection	Identified CoV Genera	Positive Bat Species (Family)	Reference	Bat Positivity Rate
Portugal ^a^ 2022	α-CoV ^a^	*Miniopterus schreibersii* * (*Miniopteridae*); *Myotis myotis* * (*Vespertilionidae*)	[38]	8.9%
Italy 2020–2022	α-CoV and β-CoV	*Hypsugo savii*, *Pipistrellus kuhlii*, *Myotis crypticus*, *Plecotus auritus*, *Pipistrellus pipistrellus* (*Vespertilionidae*)	[42]	3.1%
Italy ^a^ (Central) 2021	α-CoV ^a^	*Miniopterus schreibersii* *	Unpublished (acc.nr. ON834690, OP627105)	Not available
Lebanon ^a^ 2020	α-CoV ^a^ and β-CoV	*Miniopterus schreibersii* *	[12]	18.3%
Italy (Central–Southern) 2021	None	None	[39]	0%
Italy (Northern–Eastern) 2018	α-CoV	*Pipistrellus khulii* (*Vespertilionidae*)	[65]	undetermined
Italy ^a^ (Sardinia) 2015–2016	β-CoV ^a^	*Rhinolophus ferrumequinum* ^a^ (*Rhinolophidae*); *Plecotus auritus*, *Tadarida teniotis* (*Vespertilionidae*)	[40]	11%
Italy (Central–Southern) year not available	Not determined	Data not available	[41]	6.8%
Italy (Northwestern) ^a^ 2013–2016	α-CoV ^a^ and β-CoV ^a^	*Pipistrellus kuhlii*, *Pipistrellus pipistrellus*; *Myotis myotis*, *Myotis nattereri*, *Myotis daubentonii*, *Myotis oxygnathus*; *Plecotus auritus* (*Vespertilionidae*); *Rhinolophus ferrumequinum* ^a^	[64]	12%
France ^a^, Spain, Morocco, Tunisia 2008–2016	α-CoV ^a^ and β-CoV	*Miniopterus schreibersii* *	[21]	13.6%
Italy (Northern and Sicily, Southern) 2009–2012	α-CoV and β-CoV	*Myotis myotis*, *Myotis blythii*; *Eptesicus serotinus* (*Vespertilionidae*)	[62]	2.7%
Hungary ^a^ 2012–2013	α-CoV ^a^ and β-CoV	*Rhinolophus ferrumequinum* *; *Rhinolophus hipposideros* * (*Rhinolophidae*)	[68]	1.8%
Italy (Northern) 2010–2012	α-CoV and β-CoV	*Rhinolophus hipposideros*; *Nyctalus noctula*; *Hypsugo savii*; *P. kuhlii* (*Vespertilionidae*)	[63]	8.2%
Italy (Northern and central) ^a^ 2009	β-CoV ^a^	*Rhinolophus ferrumequinum* ^a^	[17]	3.8%
Bulgaria ^a^ 2008	α-CoV ^a^ and β-CoV ^a^	*Miniopterus schreibersii* *	[20]	40.4%

^a^ Country and geographical area for Italian strain and CoV genera linked to the CoV strains detected in this study; * only positive bat species related to CoV strains detected in this study were included in the table, while all positive bat species detected in other Italian studies have been included.

## Data Availability

The sequence data obtained in this study have been submitted to GenBank database under accession numbers PV392527-PV392538.

## Supplementary Materials

The following supporting information can be downloaded at: https://www.mdpi.com/article/10.3390/pathogens14050457/s1, Figure S1: Maximum Likelihood (ML) tree based on 287 partial RNA-dependent RNA polymerase (RdRp) gene sequences of coronaviruses (bootstrap values greater than 60 are shown). The sequences of coronavirus strain detected in this study are highlighted with black dot markings (●) indicating Alphacoronavirus strains and with black squared markings (■) indicating Betacoronavirus strains; Table S1: Details on sampling sites, number and species tested, and sample types.
