# Supplementary material for: Molecular Surveillance for Potential Zoonotic Pathogens in Troglophilus Bats: Detection and Molecular Characterization of Bat Coronaviruses in Southern Italy
Source: Pathogens. 2025 May 7;14(5):457. doi: 10.3390/pathogens14050457 (PMC12114776; doi:10.3390/pathogens14050457)
Supplement: Supplementary file 1 [file pathogens-14-00457-s001.zip › Supplementary Material Table S1.pdf]

Supplementary Material Table S1. Details on sampling sites, number and species tested, and sample types.

Sampling site	Species	Number of individuals	Samples collected			
			Oral Swabs	Urines	Faeces	Tissue samples
A - Grotta del Burrò	*Miniopterus schreibersii*	13	12	12	12	6
	Myotis capaccinii	1	1	1	1	0
	Myotis myotis	2	2	2	2	0
	Rhinolophus euryale	4	4	3	3	0
B - Grotta Chiusazza	*Miniopterus schreibersii*	55	54	0	45	0
	Rhinolophus ferrumequinum	20	20	0	19	0
	Rhinolophus hipposideros	4	4	0	4	0
C -Miniere di Castelluccio	*Rhinolophus ferrumequinum*	21	21	21	21[a]	0
	Rhinolophus hipposideros	3	3	2	3[a]	0
D - Grotta Caprara	*Miniopterus schreibersii*	23	23	22	0	0
	Rhinolophus ferrumequinum	1	1	1	0	0
	Rhinolophus euryale	2	2	2	0	0
		149	148	66	110	6

[a]Rectal swabs
