# Supplementary figures and images for: Molecular Surveillance for Potential Zoonotic Pathogens in Troglophilus Bats: Detection and Molecular Characterization of Bat Coronaviruses in Southern Italy
Source: Pathogens. 2025 May 7;14(5):457. doi: 10.3390/pathogens14050457 (PMC12114776; doi:10.3390/pathogens14050457)

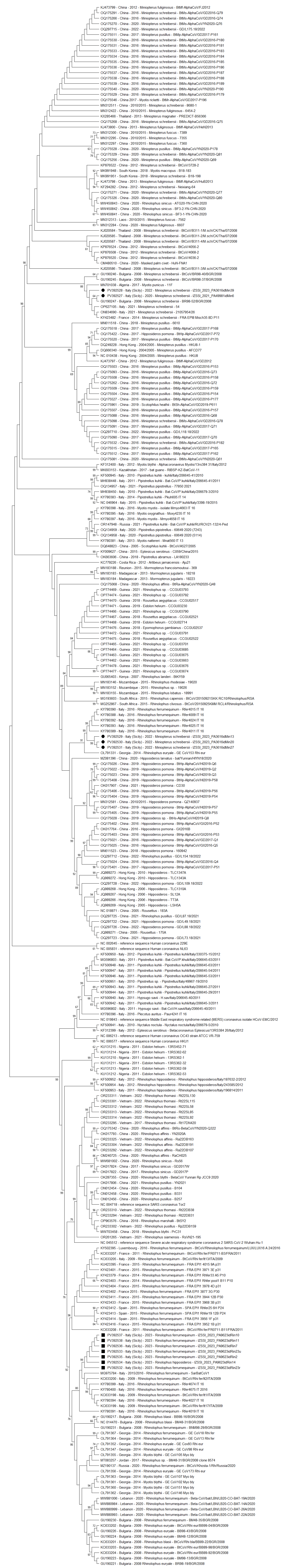

Supplement: Supplementary file 1 [file pathogens-14-00457-s001.zip › Supplementary Material Figure S1.tif]
